# Organic Phosphorus Scavenging Supports Efficient Growth of Diazotrophic Cyanobacteria Under Phosphate Depletion

**DOI:** 10.3389/fmicb.2022.848647

**Published:** 2022-03-25

**Authors:** Sophie Rabouille, Lauralie Tournier, Solange Duhamel, Pascal Claquin, Olivier Crispi, Amélie Talec, Angela Landolfi, Andreas Oschlies

**Affiliations:** ^1^Laboratoire d’Océanographie de Villefranche (LOV), CNRS, Sorbonne Université, Villefranche-sur-Mer, France; ^2^Laboratoire d’Océanographie Microbienne (LOMIC), CNRS, Sorbonne Université, Banyuls-sur-Mer, France; ^3^Department of Molecular and Cellular Biology, University of Arizona, Tucson, AZ, United States; ^4^UMR BOREA (CNRS 8067), MNHN, IRD (207), Normandie Université, Université de Caen Normandie, CREC, Luc-sur-Mer, France; ^5^GEOMAR Helmholtz Centre for Ocean Research Kiel, Kiel, Germany; ^6^CNR ISMAR, Rome, Italy

**Keywords:** *Crocosphaera watsonii*, marine, oligotrophic, dissolved organic phosphorus, phosphonates, nitrogen fixation, alkaline phosphatase

## Abstract

Considering the reported significant diazotrophic activities in open-ocean regions where primary production is strongly limited by phosphate, we explored the ability of diazotrophs to use other sources of phosphorus to alleviate the phosphate depletion. We tested the actual efficiency of the open-ocean, N_2_-fixer *Crocosphaera watsonii* to grow on organic phosphorus as the sole P source, and observed how the P source affects the cellular C, N, and P composition. We obtained equivalent growth efficiencies on AMP and DL-α-glycerophosphate as compared with identical cultures grown on phosphate, and survival of the population on phytic acid. Our results show that *Crocosphaera* cannot use all phosphomonoesters with the same efficiency, but it can grow without phosphate, provided that usable DOP and sufficient light energy are available. Also, results point out that organic phosphorus uptake is not proportional to alkaline phosphatase activity, demonstrating that the latter is not a suitable proxy to estimate DOP-based growth yields of organisms, whether in culture experiments or in the natural environment. The growth parameters obtained, as a function of the P source, will be critical to improve and calibrate mathematical models of diazotrophic growth and the distribution of nitrogen fixation in the global ocean.

## Introduction

The most important source of P to the marine environment is continental weathering, through riverine transport of particulate and dissolved phases to the coastal ocean (Delaney, 1988), to which anthropogenic sources (including sewage, livestock, or paper manufacturing) add up substantial amounts (Carpenter et al., 1998). Unlike N, P does not have a significant atmospheric gas reservoir. In the open ocean, where the impact of coastal sources is much reduced, P is supplied by aeolian dust deposition like iron (Migon et al., 2001; Mills et al., 2004) and vertical mixing from deeper layers (Galloway et al., 1996). P is thus often much more abundant in coastal areas than in the open ocean. In oligotrophic oceans, phosphate availability can be as low as a few nanomoles per liter (Wu et al., 2000; Cavender-Bares et al., 2001). Particularly low concentrations are found in the surface waters of the North Pacific and North Atlantic Oceans (Karl et al., 1997) and the Mediterranean Sea (Djaoudi et al., 2018). Brand (1991) conducted a comparative analysis of dissolved inorganic nutrient ratios and pointed out a five times higher Fe: PO_4_^3-^dissolved nutrient ratio in the Atlantic compared to the Pacific, consequent to geochemical fractionation between ocean basins (see Table 1 in Brand, 1991). The limiting character of such low concentrations imply that the dissolved, inorganic phosphorus (DIP) would ultimately control primary production in the most oligotrophic areas of the world ocean, and in particular in the Atlantic (Wu et al., 2000; Ammerman et al., 2003). This hypothesis is also further supported by analyses of dust deposition events in the Northern Atlantic (Moore et al., 2009). The general understanding of the marine P cycle is turning a corner, though, as recent advances brought to the fore a far more complex coupling between the cycles of P, N and metals than previously thought (Duhamel et al., 2021).

N_2_ fixation is the major entryway for new nitrogen in the open ocean and supports up to half of the new primary production (Karl et al., 1997; Carpenter et al., 2004; Sohm et al., 2011; Zehr, 2011), compensating for losses due to denitrification and anammox (Gruber, 2008). Through their activity of both nitrogen and carbon fixation, diazotrophic cyanobacteria represent a nitrogen source to both the dissolved and particulate pools in the marine environment. Unicellular, nitrogen-fixing cyanobacteria (UCYN) thrive in open oceans and substantially contribute to the oceanic nitrogen budget (Zehr et al., 2001; Montoya et al., 2004; Zehr and Capone, 2020). Autotrophic UCYN are largely confined to the tropics and subtropics, and commonly found in the most oligotrophic areas of the world ocean. The genus *Crocosphaera* is the only cultivated representative of the open-ocean, photo-autotrophic UCYNs and as such, represents a model organism to study the potential for nitrogen fixation in oligotrophic areas.

The ability of N_2_-fixers to grow on N_2_ explains their competitiveness in N-depleted regions, while their sensitivity to the phosphorus source is much less understood. Given that diazotrophs are usually poorer competitors than non-diazotrophic phytoplankton (Agawin et al., 2007) they should be out-competed in environments in which the DIP resource is scarce, such as the Northern Atlantic in particular. And yet, very contradicting observations prove that significant diazotrophic activities recurrently occur in the North Atlantic (Luo et al., 2012). A still unexplored route is to consider that diazotrophs may have access to another source of phosphorus to alleviate DIP depletion. Considerations of nutrients balances and the control of N_2_ fixation by the N:P ratio may, in the literature, have interchangeably used the terms phosphorus and phosphate, implicitly assuming that DIP was the unique source of phosphorus for diazotrophic growth. Phytoplanktonic organisms primarily use inorganic phosphorus (phosphate, PO_4_^3−^), which only accounts for a small fraction of the total, dissolved phosphorus found in oligotrophic oceans. But when facing low PO_4_^3−^ availability, marine microorganisms may utilize enzymes, such as alkaline phosphatase, to acquire PO_4_^3−^ from a wide diversity of P-containing, organic compounds in seawater, which together comprise the pool of dissolved organic phosphorus (DOP). DOP is composed of the following major P bond classes: P-esters [P-O-C bonds, including phosphomono-(P-O-C) and diesters (C-O-P-O-C); 80%–85%], polyphosphates (P-O-P; 8%–13%), and phosphonates (C-P; 5%–10%; Young and Ingall, 2010). Because DOP represents the largest pool of P in the oligotrophic surface ocean–typically ~80% of the total dissolved pool (Karl and Björkman, 2015), an active DOP hydrolysis in DIP limited environments would thus contribute to a local phosphorus supply for primary production. The exploitation of DOP by diazotrophs and other picophytoplankton is thus discussed as a possible explanation for their success in oligotrophic environments, especially those with limited PO_4_^3−^ availability (e.g., McLaughlin et al., 2013; Karl, 2014; Benavides and Voss, 2015). It was therefore hypothesized that DOP could participate in the control of N_2_ fixation, the marine fixed N inventory, and primary productivity (Landolfi et al., 2015, 2018; Letscher and Moore, 2015; Somes and Oschlies, 2015). But unless associated with molecular analyses that reveal which organisms are actually producing these enzymes, the *in situ* observation of such activity cannot reveal which planktonic group is responsible for it. It is also still unclear to what extent it supports primary production and diazotrophic growth. So, in comparison to growth on PO_4_^3−^, the actual ability of diazotrophs to use DOP is still poorly understood and described. The resultant efficiency in their growth, photosynthesis and diazotrophy, as well as the fate of N and P into different cellular biochemicals is thus not yet quantified.

Cyanobacteria possess genes coding for alkaline phosphatases, suggesting an ability to use some organic P-containing molecules. A *phn* gene cluster that encodes for phosphonate transport and hydrolysis proteins is for instance found in *Trichodesmium* (Dyhrman et al., 2006) and *Nodularia* (Teikari et al., 2018) and induced upon PO_4_^3−^ stress. *Crocosphaera* strains discriminate from other cyanobacteria, as they do not seem to possess the genetic baggage to hydrolyze phosphonates (Dyhrman et al., 2006). However, besides their high affinity phosphate transporters, they also present genes coding for a putative alkaline phosphatase and have the capacity to hydrolyze phosphomonoesters (Dyhrman and Haley, 2006). Some of these genes remain putative and their function has yet to be demonstrated, as well as the actual efficiency of the related DOP scavenging activity. In the present study, we focus on the latter. In order to provide a better quantification of diazotrophic growth on organic P sources when the inorganic, PO_4_ substrate becomes depleted, we investigated the growth efficiency of *Crocosphaera watsonii* on different inorganic and organic P compounds. We thus compared growth rates, but also the cellular C, N, and P stoichiometry as well as metabolic rates related to the C and N metabolism. We expected that growing on an organic source of phosphorus would require additional energy (and nitrogen) expenditure to build up a pool of active alkaline phosphatase sufficient to support phosphorus acquisition. If the acquisition of organic phosphorus is not as efficient as that of phosphate, indirect costs will add up to the unfavorable energetic budget, such as the impact of phosphorus limitation on cell functions like photosynthesis or the buildup of phosphorus containing molecules. We therefore compared metabolic activities of cultures provided either with inorganic phosphorus (phosphate) or an organic phosphorus source. For phosphorus to be the unique environmental variable to differ between treatments, and therefore prevent any bias in the results, we closely controlled the light conditions within the cultures, operating manual dilutions to keep all cultures at similar abundances, in order to ascertain that all treatments would experience the same irradiance and therefore would be provided with the same amount of light energy.

## Materials and Methods

### Culture Conditions

Experiments were carried out on unialgal cultures of the diazotrophic, marine cyanobacterium *C. watsonii* strain WH8501. Bacterial contamination was checked using flow cytometry. The contamination level remained below 2%. A culture medium devoid of phosphorus was prepared under sterile conditions, using natural, autoclaved seawater. This seawater was sampled at the Point B station (43° 41′ 10” N and 7° 19′ 00″ E), first filtered onto a 1-μm filter, and aged for at least 6 weeks. Before medium preparation, the water was filtered again through a 0.1-μm filter and autoclaved (120°C, 30 min). All macronutrients but phosphate, as well as vitamins and trace metals were added following the proportions defined by Chen et al. (1996). This common base was always prepared in one, large Nalgene bottle and once ready, was split into four different Schott bottles: a first medium was prepared with phosphate (KH_2_PO_4_) and used for control cultures grown in phosphate (thereafter PO_4_ cultures); in the three other media, an organic phosphorus source was added instead of phosphate, such that final concentrations of P in all culture media were all 50 μmol P L^−1^. The chosen organic sources were DL-α-glycerophosphate (thereafter Alpha cultures), adenosine mono phosphate (thereafter AMP cultures) and phytic acid.

One liter, triplicate cultures of each treatment were prepared and equipped with tubing mounted through the lid to allow for sampling and air bubbling. The sampling tubing was closed with a clamp to prevent any back-flow into the culture. The air tubing was capped with a 0.2-μm air filter to prevent contamination through the air intake. Cultures were gently bubbled and maintained homogeneous using a magnetic stirrer, in addition to manual, stirring operation prior to each sampling. Cultures were maintained at a temperature of 27°C, previously identified as being optimal for growth (Webb et al., 2009) and exposed to an incident irradiance of 200 μE m^−2^ s^−1^ using fluorescent tubes (white light). The light:dark cycle was set to a square, 12 h light and 12 h dark regime.

Cultures were initially inoculated in their respective medium (i.e., containing either DIP or DOP) using the same seed culture for all treatments, and then operated as a fed-batch mode: each culture flask was refreshed periodically in their respective medium, depending on the increase in population density, to insure (i) the maintenance of cells in an exponential state of growth, (ii) prevent them from reaching too high a cell density, which would have affected the actual irradiance perceived within the cultures, and (iii) maintain similar densities in all the cultures and therefore insure that all the different treatments experience the same irradiance, which is a crucial point of this experiment. The growth rate in between dilution phases was derived from the cell abundance monitoring. Cultures were pre-acclimated to their respective conditions for over 45 days, to ensure that metabolic rates and physiological properties were not the remnant of their previous dynamics on phosphate. Cultures were monitored for cell counts on a daily basis and sampled for biochemical parameters during the exponential phase.

The standard parameters recorded on the cultures included cell numbers, cellular contents in carbon, nitrogen, and phosphorus, soluble reactive phosphorus (SRP), total dissolved phosphorus (TDP), particulate organic phosphorus (POP), dissolved organic nitrogen (DON), ammonium (NH_4_), nitrite (NO_2_), and nitrate (NO_3_). We also monitored alkaline phosphatase activity and the diel dynamics of photosynthetic efficiency and nitrogen fixation activity.

Cell counts were performed daily using a Beckman Multisizer Coulter Counter, on samples diluted 100 times with filtered seawater. The average cell size in the population was derived from the size spectrum provided by the Coulter upon each count of the population.

The following convention was used regarding time: the day of inoculation at the onset of the light cycle was taken as the time zero for each experiment. Therefore, experimental days are synchronized with the light cycle; e.g., day 15.0 is the 15th day into the experiment at the onset of the light and day 15.5 is 12 h into the 15th day, which is the light to dark transition.

### Biochemical Analyses

#### Particulate Organic Phosphorus and Dissolved Phosphorus (SRP and TDP)

Volumes of 22 ml were sampled daily on each culture and filtered on combusted (4.5 h at 450°C) GFF filters. Filters were used to quantify POP and filtrates were used for the analysis of dissolved phosphorus. Samples were processed by combustion, according to Strickland and Parsons (1972). Filtrates were divided in two 11 ml subsamples and stored at −80°C until analysis for SRP and TDP. Their concentrations were determined by spectrophotometry according to Murphy and Riley (1962). A calibration curve was prepared from a 1,000 nmol⋅l^−1^ solution of KH_2_PO_4_.

#### Particulate Organic Carbon (POC) and Nitrogen (PON)

Volumes of 15 ml were sampled daily on each culture and filtered on combusted (4.5 h at 450°C) GFC filters. Filters were used for the analysis of the particulate material and filtrates were used to quantify the dissolved fraction. POC and PON were analyzed using a Flash Elemental Analyzer (Thermo Fisher Scientific), according to the method described in Moran et al. (1999), with the standard acetanilide nicotinamide. Cell contents were estimated using the cell abundance measured at the time of sampling.

#### Dissolved Organic and Inorganic (NO_3_^−^, NO_2_^−^, and NH_4_^+^) Forms of Nitrogen

Filtrates from POC-PON samples were filtered a second time on GFF filters to respect the usual convention of a 0.7-μm porosity to discriminate between particulate and dissolved fractions, and then placed in pre-acidified Falcon tubes and stored at −80°C until analysis. Filtrates from replicates #1 were used to quantify DON; they were processed according to Pujo-pay and Raimbault (1994) and analyzed using a Technicon autoanalyzer. Filtrates from replicates #2 were used to quantify NO_3_^−^ and NO_2_^−^. Samples were measured by colorimetry according to Raimbault et al. (1990), using a Technicon autoanalyzer. For all these dissolved fractions, standard solutions of NO_3_^−^ and NO_2_^−^ at 10 μmol⋅l^−1^ and blanks were prepared using a 38 g⋅l^−1^ NaCl solution, matching the salinity of the culture medium. Filtrates from replicates #3 were used to quantify NH_4_^+^ and measured by fluorimetry according to Holmes et al. (1999), using an Alliance analyzer (FUTURA). A calibration curve with concentrations ranging from 0 to 5,000 nmol l^−1^ was prepared in a 35 g⋅l^−1^ solution of NaCl.

#### Nitrogenase Activity

We measured nitrogenase activity in a preliminary experiment carried out on turbidostats cultures maintained in similar conditions as the present experiments. Nitrogenase activity was recorded following the method described in Staal et al. (2001) and using the same setup as in Dron et al. (2012). Fourty milliliter culture from one culture was sampled at the end of the light phase and filtered onto a GFC filter. The filter was placed into an air-tight incubation chamber, overlying a layer of culture medium to maintain the filter fully moist. This chamber had a temperature controlled at 27°C and a flux of N_2_/O_2_/C_2_H_2_ gas mixture (70%/20%/10%). The nitrogen and oxygen gas tanks used contained 400 ppm CO_2_. The gas flow rate was controlled using Brooks mass flow controllers and set to 1 L⋅h^−1^. The GC operated one acquisition every 10 min. Ethylene production rates were converted into nitrogen fixation rates (fmol N_2_ fixed cell^−1^ day^−1^) considering a conversion factor of four to account for the stoichiometric difference between the reduction of acetylene and that of dinitrogen (Capone, 1993; Staal et al., 2001).

#### Respiration Rates

Fifteen milliliter culture from one culture was sampled and transferred to an air-tight incubation beaker equipped with a Fibox (PreSens) sensor spot, removing any headspace to prevent exchanges between the liquid and gas phases. A Fibox® 4 fiberoptics was connected to the beaker and this setup was immersed in the dark in a temperature-controlled water bath at 27°C. An automated monitoring of oxygen concentrations was immediately started. Acquisitions were done every 5 s from 20 to 30 min. Due to the availability of a single incubation beaker, oxygen measurements were repeated in time, every 20 to 30 min, over the entire dark phase.

#### Photosynthetic Efficiency

Photosynthetic activity was monitored over a day at 1 h intervals using a Multicolor PAM (Walz, Effeltrich, Germany). The maximum quantum efficiency of photosystem II (PSII) charge separation (Fv/Fm) was measured without dark adaptation to prevent state transitions from occurring, following the protocol described in Rabouille and Claquin (2016). The sample was excited by a weak blue light (1 μmol photons m^−2^ s^−1^, 470 nm, frequency 0.6 kHz) to record minimum fluorescence (*F*_0_). Maximum fluorescence (*F*_m_) was measured following a saturating light pulse (0.6 s, 1,700 μmol photons m^−2^ s^−1^, 470 nm). Light-response (PI) curves were recorded on each sample. These were exposed to 20 irradiances (I) for 1 min each, covering a range from 0 up to about 1,900 μmol photons m^−2^ s^−1^. The equations proposed by Genty et al. (1989) were used to calculate the effective quantum efficiency of PSII in the dark (*F*_v_/*F*_m_) and at each irradiance (*F*_v_^′^/*F*_m_^′^). These fluorescence parameters were used to derive the relative electron transport rate (rETR, arbitrary unit) at each irradiance:


$$\text{rETR}={\text{F}}_{\text{V}}\prime /{\text{F}}_{\text{m}}\prime \cdot I$$

Results were fitted using the mathematical formulation proposed by Bernard and Rémond (2012) to derive the optimal rETR value (rETR_opt_) observed in each PI curve:


$$\mathrm{rETR}\left(I\right)\;=\;{\mathrm{rETR}}_{\max}\cdot \frac{I}{I+\frac{{\mathrm{rETR}}_{\max}}{\alpha }\cdot {\left(\frac{I}{I_{opt}}-1\right)}^{2}}$$

#### Alkaline Phosphatase Activity

Alkaline phosphatase activity was monitored according to Jauzein et al. (2010) and Duhamel et al. (2011). Five milliliter samples were taken from each culture replicate; 3,920 μl were transferred into a test tube, to which 80 μl of a 500 μmol⋅l^−1^ MUF-P reagent were added, to obtain a final volume of 4 ml. Fluorescence was measured using a fluorimeter (CARY Elipse Varian) with a 355-nm excitation wavelength and a 453 nm emission wavelength. The instrument blank was calibrated using milli-Q water. Blank measurements were obtained by reading the samples before adding the MUF-P reagent. A first reading was performed immediately after adding the MUF-P (*t*_0_), then at *t*_0_ + 30 min, *t*_0_ + 1 h, and *t*_0_ + 3 h. The standard curve was obtained using a MUF solution with concentrations ranging from 0 to 1,000 μmol⋅l^−1^.

#### Statistical Analyses

In the following, we present measured cell sizes, rates, and concentrations, and we compare the average values obtained among the three treatments. The statistical significance was tested using an ANOVA test for equal means with a *post-hoc*, Tukey test run over the three data sets; we test the rejection of the null hypothesis at the significance level of 5%.

## Results

We observed the growth rate and biomass composition of cultured populations of *C. watsonii* grown in media that only differ by their P source. Each triplicate culture was provided with one of the following: phosphate (taken as a reference) or DL-α-glycerophosphate or adenosine mono phosphate or phytic acid.

**Figure 1:**
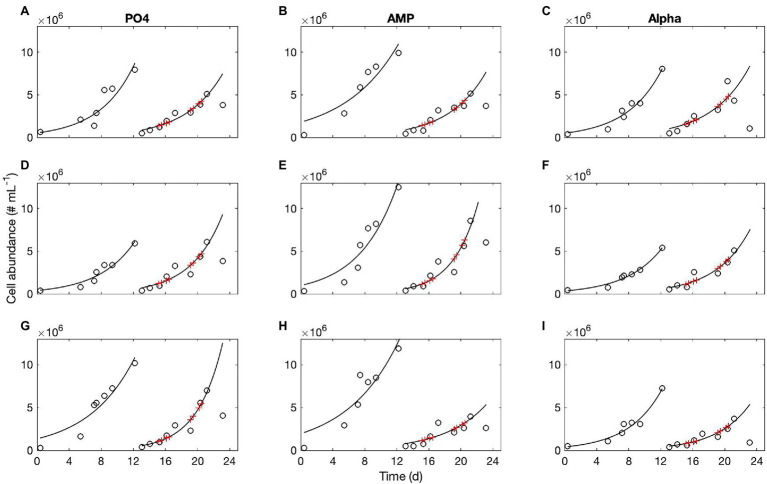
Typical growth curves obtained in three replicates of the PO_4_ cultures **(A,D,G)**, AMP cultures **(B,E,H)**, and Alpha cultures **(C,F,I)**. Open symbols, population abundance in cell numbers per ml; red crosses indicate when POP samples were taken for analyses. In this example, all flasks were diluted on day 12 with their respective medium containing either an inorganic (PO_4_) or organic [mono phosphate adenosin (AMP) or DL-α-glycerophosphate (Alpha)] source of phosphorus for growth.

Concentrations of biochemical species are provided with standard deviations; note that these do not correspond to analytical triplicates but to the measurements taken in the three culture replicates, which explains why variability may be large. A very small difference in the initial concentrations of cells, for instance, may lead to notable discrepancies in the final population abundance and so in the bulk elemental concentrations. This variability does not affect the population dynamics and growth rates as the growing cultures remain non limited throughout the sampling period. We recorded *n* = 9 growth curves on the PO and AMP cultures, and 11 on the Alpha cultures. An example of population monitoring is shown in Figure 1. The corresponding cell sizes are shown in the Supplementary Material.

### Population Dynamics

Population dynamics was monitored throughout the entire experiment and the exponential growth rates determined over several, consecutive growth phases, in order to obtain several estimations of the growth rate, replicated in time. The mean exponential growth rate estimated in the PO_4_ culture is μ_PO__4_ = 0.23 ± 0.05 day^−1^ (*n* = 9), with a maximum of 0.31 ± 0.06 day^−1^. The mean, exponential growth rate estimated in the AMP culture is μ_AMP_ = 0.21 ± 0.05 day^−1^ (*n* = 9) with a maximum of 0.32 ± 0.08 day^−1^. The mean exponential growth rate estimated in the Alpha culture is μ_Alpha_ = 0.22 ± 0.03 day^−1^ (*n* = 11) with an observed maximum of 0.23 ± 0.007 day^−1^. Cultures grown in phytic acid survived, showing a roughly constant cell abundance over time (it was not possible to fit a growth curve), indicating that the gross growth rate is slightly positive and just sufficient to compensate for the population mortality, leading to a zero net growth rate. The slow growth in the culture did not generate sufficient biomass density for biochemical analyses. Therefore, in the following, only results for the PO_4_, AMP, and Alpha cultures are shown. The exponential growth rates observed in these three treatments do not significantly differ from each other at the significance level of 5% (*p* = 0.443). That is, while *C. watsonii* barely survives on phytic acid, it grows equally well on two other sources of organic phosphorus (AMP and DL-α-glycerophosphate) compared to phosphate.

The average cell size measured during the experiments is presented in Table 1. Cultures grown on AMP and Alpha are significantly larger than the PO_4_ cultures (*p* = 8.55·10^−4^).

**Table 1:** Average cell sizes and their respective standard deviation measured in exponentially growing cultures of *Crocosphaera watsonii* provided with either an inorganic (KH_2_PO_4_) or organic [mono phosphate adenosin (AMP), DL-α-glycerophosphate (Alpha) or phytic acid] source of phosphorus for growth.

	Cell size (μm ± sd)
PO_4_	3.37 ± 0.10 (*n* = 45)
AMP	3.46 ± 0.13 (*n* = 46)
Alpha	3.45 ± 0.11 (*n* = 43)
Phytic acid	3.83 ± 0.55

*The value in between bracket is the number of samples used to estimate this average*.

### Cellular Pools

#### Particulate Organic Carbon and Nitrogen Content

The particulate carbon and nitrogen contents in the cell biomass are shown in Table 2. The overall N content per cell is higher in the AMP cultures, with statistical significance (*p* = 0.0035), while the C content presents no significant difference between cultures. As samples were taken in the beginning and at the end of the light phase, an absence of statistical significance may be related to the wide fluctuations in cell stoichiometry observed in this strain over the diel cycle (Dron et al., 2012), leading to large deviations around the mean. We distinguished the C and N contents between these two sampling times. We find neither statistical difference at the 5% level in the N or C content among the samples taken in the early light phase, nor among those taken in the late-light phase. The N and C contents in the early (resp. the late) light phase are thus statistically similar. We also compared the difference between the amounts incorporated in cells during the light cycle (Table 2). The average amount of nitrogen (mean ΔN) acquired over the dark phase appears larger in the PO_4_ culture, while the average amount of carbon (mean ΔC) incorporated during the light phase is larger in the AMP culture. However, due to the rather large standard deviations, the differences between the ΔN or the ΔC are not statistically significant. We also estimated the C and N contents per unit of cell volume (fmol C μm^−3^ and fmol N μm^−3^, shown in Table 3) to avoid a possible bias due to differing cell sizes. A comparison of the volumetric contents still returns that differences between the ΔN or the ΔC expressed per volume unit are not statistically significant.

**Table 2:** Mean N (fmol N cell^−1^) and C (fmol C cell^−1^) content in cultures of *Crocosphaera watsonii* provided with either an inorganic [phosphate (PO_4_)] or organic [mono phosphate adenosin (AMP), DL-α-glycerophosphate (Alpha)] source of phosphorus for growth.

Culture	PO_4_	AMP	Alpha
Mean N content (# data points)			
Early light phase	81.2 ± 18.2 (18)	81.0 ± 34.6 (17)	67.4 ± 27.0 (18)
Late light phase	55.8 ± 24.2 (18)	69.8 ± 30.9 (16)	51.7 ± 24.2 (18)
Mean ΔN incorporated per night	25.4 ± 33.1 (18)	12.2 ± 42.3 (16)	15.8 ± 34.2 (18)
Mean C content (# data points)			
Early light phase	463.6 ± 163.4 (18)	497.8 ± 181.7 (17)	440.7 ± 137.8 (18)
Late light phase	499.1 ± 145.0 (16)	646.1 ± 317.7 (17)	529.0 ± 269.2 (18)
Mean ΔC incorporated per day	56.4 ± 206.0 (16)	148.3 ± 325.0 (17)	88.31 ± 240.24 (18)

*N and C contents were measured in the early and late light phase. The value in between bracket is the number of samples used to estimate this average*.

**Table 3:** Mean N (fmol N μm^−3^) and C (fmol C μm^−3^) content in cultures of *Crocosphaera watsonii* provided with either an inorganic [phosphate (PO_4_)] or organic [mono phosphate adenosin (AMP), DL-α-glycerophosphate (Alpha)] source of phosphorus for growth. N and C contents were measured in the early and late light phase.

Culture	PO_4_	AMP	Alpha
Mean N content (# data points)			
Early light phase	4.2 ± 0.9 (18)	4.1 ± 1.7 (17)	3.3 ± 1.3 (18)
Late light phase	2.9 ± 1.3 (18)	3.5 ± 1.6 (16)	2.5 ± 1.2 (18)
Mean ΔN incorporated per night	1.3 ± 1.7 (18)	0.6 ± 2.1 (16)	0.8 ± 1.7 (18)
Mean C content (# data points)			
Early light phase	24.0 ± 8.5 (18)	25.0 ± 9.1 (17)	21.4 ± 6.7 (18)
Late light phase	25.8 ± 7.5 (16)	32.5 ± 16.0 (17)	25.7 ± 13.1 (18)
Mean ΔC incorporated per day	2.9 ± 10.7 (16)	7.5 ± 16.3 (17)	4.3 ± 11.7 (18)

**Figure 2:**
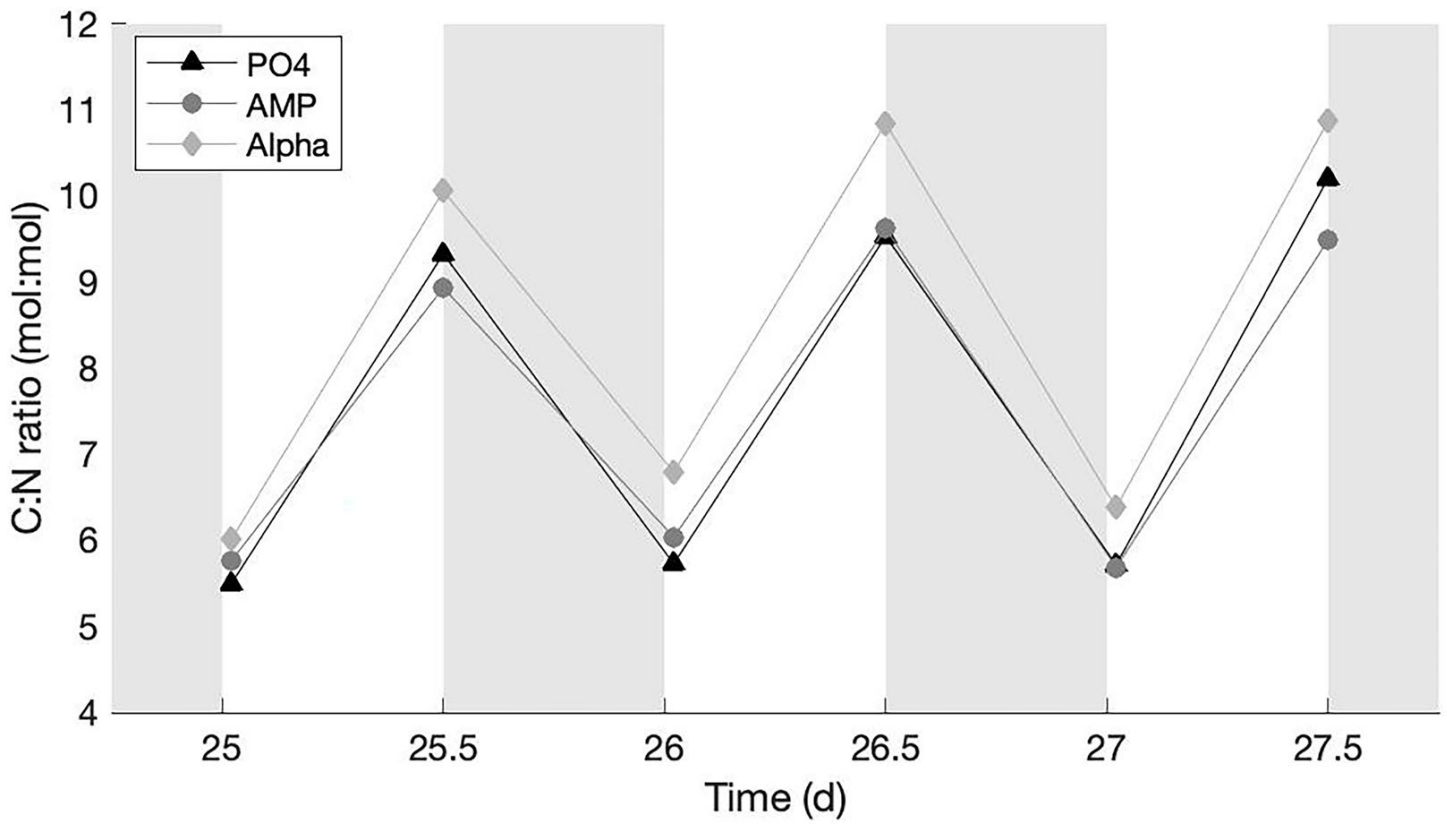
C:N ratios in cultures provided with either an inorganic (PO_4_; triangles) or organic [mono phosphate adenosin (AMP; circles) or DL-α-glycerophosphate (Alpha; diamonds)] source of phosphorus for growth. The grey, shaded area represents the dark phase.

The average C:N ratio in all treatments presents a typical fluctuation (Gallon, 1992; Sherman et al., 1998; Dron et al., 2012, 2013) with lower values in the early-light (at the end of the N_2_ fixation period) and higher values in the late-light phase (Figure 2). The PO_4_ culture observed fluctuations with an amplitude that is intermediate between the AMP culture (the lowest amplitude) and Alpha (the largest amplitude). All values are above the Redfield C:N ratio, instead of fluctuating around it. We find no statistical difference among the early light, average C:N ratios in the different treatments. Among the late light samples, the average C:N ratio is significantly higher in the Alpha culture than in the AMP; but the C:N ratio in neither culture grown on DOP significantly differs from that in the reference PO_4_ culture.

#### Particulate Organic Phosphorus Content

Since we observed some differences in cell size between treatments, we normalized the cellular phosphorus content both per cell (Table 4) and per unit of cell volume (Table 5). When considering all samples, the average POP content is 7.17 ± 1.33, 9.43 ± 2.37, and 5.98 ± 2.22 fmol P cell^−1^ in the PO_4_, AMP, and Alpha cultures, respectively. Normalizing per unit of cell volume, the POP content is 0.046 ± 0.010, 0.055 ± 0.015, and 0.034 ± 0.014 fmol P μm^−3^ in the PO_4_, AMP, and Alpha cultures, respectively. That is, the volumetric phosphorus content in the AMP and Alpha cultures is 120% and 73% of the average volumetric content in the PO_4_ cultures. The reference culture thus shows an intermediate phosphorus content compared to the two cultures grown on DOP. The per cell POP content in the AMP culture is significantly higher than the other two at the 5% level, while it is similar in the PO_4_ and Alpha cultures. But it can be expected that being bigger, cells in the AMP cultures contain more material. When considering the volumetric contents, cells in the AMP and PO_4_ cultures then show similar POP contents while that in Alpha is statistically lower from both AMP and PO_4_.

**Table 4:** Particulate phosphorus content normalized per cell, measured in exponentially growing cultures of *Crocosphaera watsonii* provided with either an inorganic (PO_4_) or organic [mono phosphate adenosin (AMP) or DL-α-glycerophosphate (Alpha)] source of phosphorus for growth.

	PO_4_	AMP	Alpha
Average cell abundance (10^6^ cells ml^−1^) over the sampling period	2.81	2.74	2.86
Average P content (all samples pooled; fmol P cell^−1^)	7.17 ± 1.33 (*n* = 21)	9.43 ± 2.37 (*n* = 21)	5.98 ± 2.22 (*n* = 21)
Average P content in the early light phase (fmol P cell^−1^; 1st morning is an outlier)	8.25 ± 1.14 (*n* = 9)	10.23 ± 2.35 (*n* = 9)	6.94 ± 2.22 (*n* = 9)
Average P content in the late light phase (fmol P cell^−1^)	6.37 ± 0.77 (*n* = 12)	8.84 ± 2.30 (*n* = 12)	5.27 ± 2.02 (*n* = 12)
P decrease in the light (fmol P cell^−1^)	2.03 ± 0.82	2.10 ± 1.72	1.96 ± 1.22
P decrease in the light (% of the early light phase quota)	24.62%	20.53%	28.24%
P increase in the dark (fmol P cell^−1^)	1.88 ± 1.53	1.25 ± 2.00	1.74 ± 1.43
P increase in the dark (% of the late light phase quota)	29.51%	14.14%	33.02%

**Table 5:** Particulate phosphorus normalized per cell volume, measured in exponentially growing cultures of *Crocosphaera watsonii* provided with either an inorganic (PO_4_) or organic [mono phosphate adenosin (AMP) or DL-α-glycerophosphate (Alpha)] source of phosphorus for growth.

	PO_4_ (C2)	AMP (C1)	Alpha (C3)
Average cell volume (um^3^) over the sampling period	20.04 ± 0.001	21.69 ± 0.001	21.50 ± 0.001
Average P content (all samples pooled; fmol P um^−3^)	0.046 ± 0.010 (*n* = 24)	0.055 ± 0.015 (*n* = 21)	0.034 ± 0.014 (*n* = 21)
Average P content in the early light phase (fmol P um^−3^)	0.0621 ± 0.0180 (*n* = 12)	0.0599 ± 0.0147 (n = 9)	0.0530 ± 0.0244 (*n* = 12)
Average P content in the late light phase (fmol P um^−3^)	0.0404 ± 0.0060 (*n* = 12)	0.0507 ± 0.0144 (*n* = 12)	0.0285 ± 0.0122 (*n* = 12)
P decrease in the light (fmol P um^−3^)	0.0217	0.0092	0.0245
P decrease in the light (% of the early light phase quota)	34.94%	15.36%	46.22%
P increase in the dark (fmol P um^−3^)	0.0118 ± 0.0112	0.0077 ± 0.0159	0.0112 ± 0.0104
P increase in the dark (% of the late light phase quota)	29.2%	15.2%	39.3%

POP contents also show a diel trend with higher values at the onset of the light and lower at the onset of the dark (Tables 4 and 5) and this fluctuation may affect the statistical comparison of the different treatments. Within the early light samples, the POP contents expressed per cell are similar in the phosphate and Alpha cultures while the AMP culture differs from the other two at the 5% level. The same differences are observed within the late light samples. When normalizing per volume of cell, none of the POP contents in the early light samples differs from the others; and in late light samples AMP and PO_4_ remain similar while the Alpha culture shows lower contents compared to the other two. The differences observed on the daily averaged values are thus also found when separately comparing the early light and early dark samples. We then estimated the POP decrease in the light phase and the POP increase in the dark phase. Note that the latter avoids the bias that cell division introduces during the light phase. Overall, the AMP culture shows the highest P content and the smallest relative increase in the dark, while the Alpha culture shows the lowest P content but a proportionately larger relative increase in the dark (Tables 4, 5).

### Alkaline Phosphatase Activity

Alkaline phosphatase activity is normalized using cell abundance and the duration of the incubation, and averaged from the triplicate samples. It is thus expressed in pmol (P hydrolyzed) cell^−1^ h^−1^ (Table 6A) and shows no significant difference between the treatments. An important fact is that activities were always recorded at the same time on all cultures, but over different days and times in the light cycle. This data set indicates that the activity is not linear over a 3-h incubation: the estimated rate is not the same depending on whether we calculate it over 30 min, 1 or 3 h. Further, there also seems to be a diel trend in each culture: the recorded activities tend to be stronger in the early light phase compared to the late light phase. The alkaline phosphatase may thus not be produced monotonically over the light cycle. For all these reasons, these absolute activities cannot be pooled within each treatment. In order to remove any bias due to temporal dynamics in the light cycle or to incubation time, the same data were expressed as relative activities (Table 6B). Normalization is performed relatively to Alpha, which always shows the highest activity. Activities in AMP and PO_4_ at any given time is thus expressed relatively to the activity in Alpha measured at the same moment. This allows comparing all data and in particular records from different incubation times or different sampling hours. Here, the three cultures show significantly different activities at the 5% level. Alkaline phosphatase activity in the PO_4_ culture is very low while it is much higher in the two DOP-grown cultures. Last, activity in the Alpha culture is also more than twofold that observed in AMP.

**Table 6:** Alkaline phosphatase activity measured in exponentially growing cultures of *Crocosphaera watsonii* provided with either an inorganic (PO_4_) or organic [mono phosphate adenosin (AMP) or DL-α-glycerophosphate (Alpha)] source of phosphorus for growth.

Incub. time (h)	PO_4_	AMP	Alpha
**A**	pmol (P hydrolyzed) cell^−1^ h^−1^		
0.5	0	0.066	0.406
0.5	0	0.070	0.263
0.5	0.004	0.044	0.077
1	0.004	0.102	0.505
1	0.010	0.051	0.111
3	0.005	0.096	0.347
3	0.015	0.054	0.123
**B**	**Relative activity**		
0.5	0	16.26	100
0.5	0	26.62	100
0.5	5.19	57.14	100
1	0.81	19.68	100
1	9.00	45.95	100
3	1.44	27.67	100
3	12.20	52.85	100

***(A)** Activity per cell [pmol (P hydrolyzed) cell^−1^ h^−1^]. **(B)** The same records but expressed in proportion to the activity measured in the Alpha culture. Note that the PO_4_ culture is not taken as reference here because of the two null values recorded; so we instead normalized to the culture with the highest activity*.

### Nutrient Concentrations in the Cultures

#### SRP and TDP Concentrations

We measured SRP and TDP concentrations in the cultures after 19 days of growth. The reference cultures, grown on phosphate, show the highest levels of SRP with a concentration of 23.93 ± 4.15 μmol P L^−1^. The lowest SRP concentration is found in the Alpha cultures (4.71 ± 0.90 μmol P L^−1^) while the AMP cultures show an intermediate concentration between the former two (14.01 ± 1.75 μmol P L^−1^). Nevertheless, SRP levels provide a partial information as they correspond to the inorganic fraction and thus do not indicate how much DOP actually remains in the AMP and Alpha cultures. In the following, we thus compared the levels of the total, dissolved phosphorus in the medium, that inform on how much dissolved phosphorus is still potentially available for growth.

After 19 days of growth, the reference cultures, grown on phosphate, show a TDP concentration of 28.43 ± 3.38 μmol P L^−1^, whose range agrees well with the SRP level. The AMP cultures show a TDP level of 16.98 ± 2.90 μmol P L^−1^, which closely corresponds to the SRP level, suggesting that all the organic phosphorus in that culture was hydrolyzed and converted to phosphate by the alkaline phosphatase. In the Alpha cultures, the TDP concentration is 30.84 ± 6.07 μmol P L^−1^, suggesting that most of the remaining phosphorus is still organic.

#### Compared Dynamics of the Total Dissolved (TDP) and Particulate (POP) Phosphorus Pools

**Figure 3:**
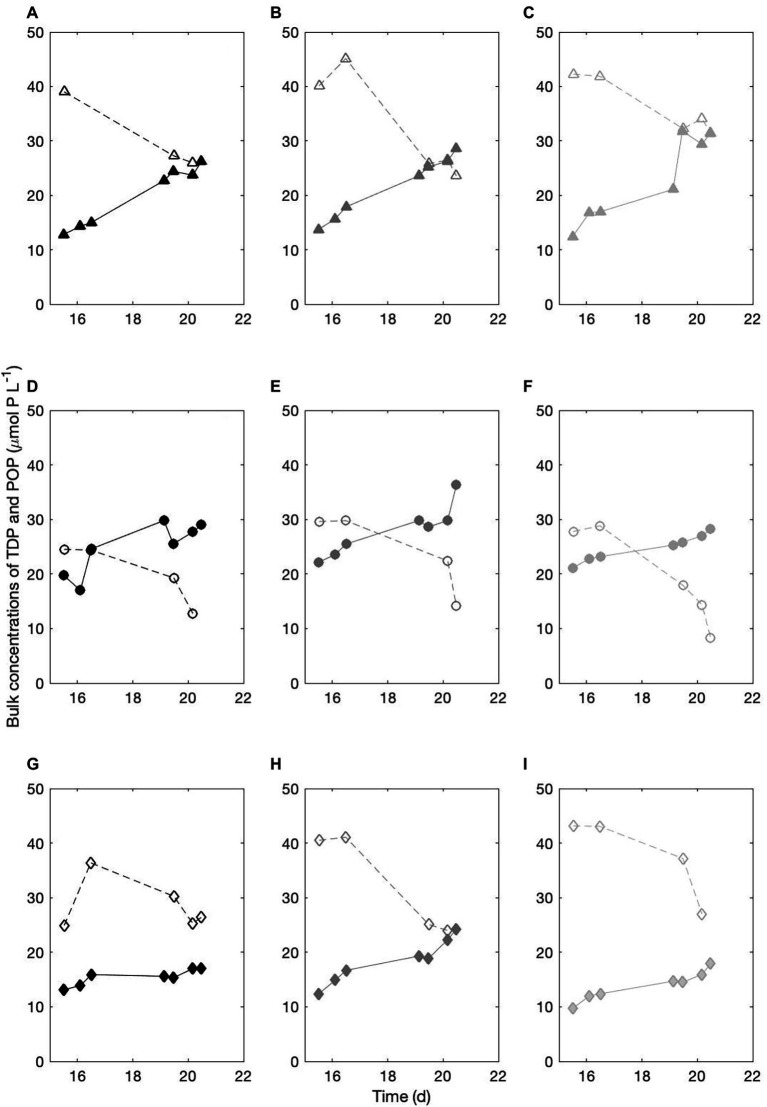
Temporal dynamics of the bulk, total dissolved and particulate phosphorus pools. **(A-C)** three replicates of the PO_4_ culture; **(D-F)** three replicates of the AMP culture; **(G-I)** three replicates of the Alpha culture. Open symbols, TDP concentrations in μmol P L^−1^. Closed symbols, POP concentrations in μmol P L^−1^.

We compared the TDP dynamics to that of the phosphorus in the cell biomass (POP). Figure 3 presents the TDP (open symbols) and POP (closed symbols) concentrations in μmol P L^−1^ measured over 5 consecutive days. In all treatments, the biomass increase is matched with a consistent decrease in the TDP in the culture medium, indicative of a clear consumption of the DOP compounds in the AMP and Alpha cultures.

#### Dissolved Nitrogen Concentrations

##### Dissolved Inorganic Nitrogen

The culture medium was prepared without any source of dissolved inorganic nitrogen (DIN). Samples were taken to verify that DIN was absent from the cultures. NH_4_^+^ levels are mostly below 2 to 4 μmol L^−1^. Nitrate and nitrite remain mostly below 2 μmol L^−1^. As expected, DIN levels remain negligible in proportion to the other nutrients over the course of the experiment.

##### Dissolved Organic Nitrogen

We found significant amounts of exuded, organic nitrogen in the culture. At the end of the sampling period (on day 27), DON levels are 61, 152, and 36 μmol N L^−1^ in the PO_4_, AMP, and Alpha cultures, respectively. The levels of bulk nitrogen biomass formed in the cultures over the same period are 231, 299, and 227 μmol PON L^−1^, respectively. Cells thus partition their gross nitrogen acquisition as follows: in the PO_4_ cultures, 80% is incorporated in the biomass and 20% exuded; in the AMP cultures, 66% is incorporated in the biomass and 34% exuded; in the Alpha cultures, 86% is incorporated in the biomass and 14% exuded.

#### Nitrogen Fixation Rates

**Figure 4:**
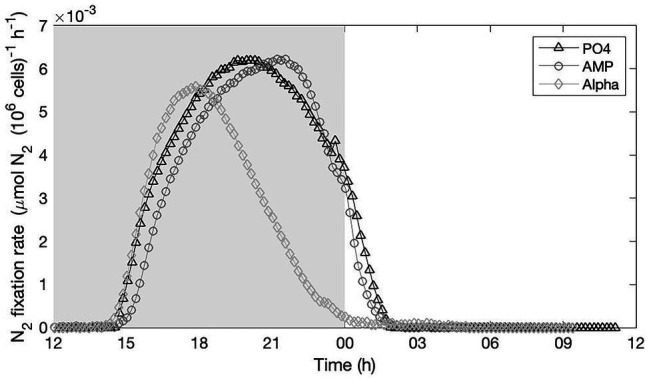
Nitrogenase activity monitored on-line in cultures provided with either an inorganic (PO_4_; triangles) or organic [mono phosphate adenosin (AMP; circles) or DL-α-glycerophosphate (Alpha; diamonds)] source of phosphorus for growth. The grey, shaded area represents the dark phase.

We recorded nitrogenase activity overnight in the three culture conditions, starting before the onset of the dark and until after the onset of the light the following day (Figure 4). A unique record per culture is not sufficient to quantitatively compare the efficiency and yield of nitrogen fixation between the treatments; but results clearly show that nitrogenase was active in all three culture treatments with rather similar temporal dynamics and maximum efficiency. We concomitantly recorded the respiration rate in the three treatments over the dark phase. No sensible difference appears between the treatments, which show comparable temporal dynamics in the rate of oxygen consumed (see Supplementary Material).

#### Photosynthetic Efficiency

**Figure 5:**
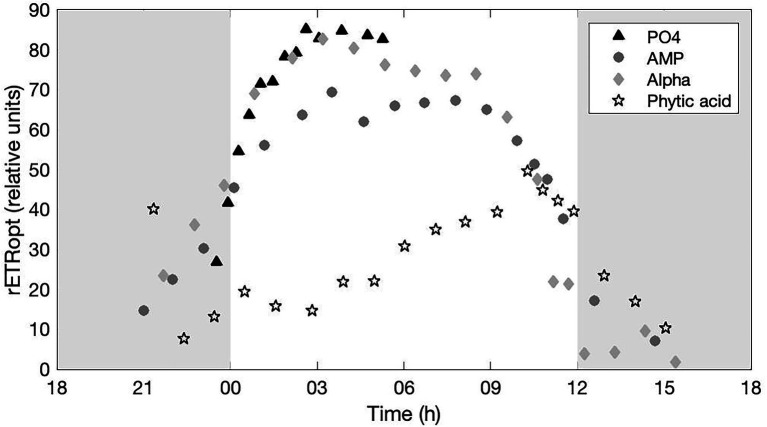
Optimal, relative electron transport rate (rETR_opt_) monitored in cultures provided with either an inorganic (PO_4_; triangles) or organic [mono phosphate adenosin (AMP; circles), DL-α-glycerophosphate (Alpha; diamonds) or phytic acid (Phytic acid; pentagrams)] source of phosphorus for growth. The grey, shaded area represents the dark phase.

The rETR expresses the instantaneous efficiency of PSII under the current incident irradiance. The changes in time of the optimal rETR (rETR_opt_) observed in each treatment are drawn on Figure 5. A clear and very similar temporal dynamics is observed in the PO_4_, AMP, and Alpha cultures, with a maximal PSII efficiency around the mid-light phase and relatively comparable maximal efficiencies. Replicated acquisitions over several days would be necessary to assess whether the slightly lower maximal efficiency in the AMP culture has any statistical significance. In contrast, the cultures grown on Phytic acid show a clearly impaired photosynthetic efficiency.

## Discussion

It has long been acknowledged that nitrogen fixation being the only biological entryway of new nitrogen into the open ocean, it constitutes a main driver of oceanic primary production (Capone et al., 1997; Karl et al., 1997). Yet, how much N_2_ is actually fixed per year is still very uncertain due to the paucity of direct measurements and also to methodological challenges (Großkopf and LaRoche, 2012; Landolfi et al., 2018). Biogeochemical models could supplement the *in situ* measurements for more exhaustive estimations but appropriate calibration data are still missing for the actual, current contribution of nitrogen fixers to the carbon (C), nitrogen (N) and phosphorus (P) cycles to be adequately evaluated in these models. Simulating realistic marine N_2_ fixation patterns is a challenge that requires experimental measurements of key processes and key parameters of matter and energy fluxes related to diazotrophic growth. In this regard, culture approaches on isolated strains can provide relevant data on specific, metabolic rates.

A major control of N_2_ fixation by PO_4_^3−^ availability has been suggested (Sañudo-Wilhelmy et al., 2001) and its excess relative to NO_3_^−^ is often the driver of diazotrophic growth in global biogeochemical models (Deutsch et al., 2007; Weber and Deutsch, 2012; Landolfi et al., 2013). The consumption of organic phosphorus is an important, yet still overlooked, feature in diazotrophic cyanobacteria that could be essential to their development in oligotrophic oceans. PO_4_^3−^ stress induces alternative, organic P (DOP) scavenging strategies in diazotrophs (Mulholland et al., 2002; Dyhrman et al., 2006). DOP use may thus be a strategic adaptation to PO_4_^3−^ limiting conditions common to many oligotrophic environments but is not systematic in all marine cyanobacteria and also depends on the compounds structure. Following these pioneering works, genes encoding for alkaline phosphatase were shown to be expressed in populations of both colonial (Orchard et al., 2009) and unicellular diazotrophs (Dyhrman and Haley, 2006), and up-regulated under P starvation. The assimilation of DOP is most probably costly in terms of energy and nitrogen. Therefore, the ability of diazotrophs to fix N_2_ might be a necessary advantage to exploit the complex constituents of the DOP pool, bearing in mind that such activity may also increase the cellular carbon demand.

Here, we analyze the capacity of the N_2_ fixer *C. watsonii* WH8501 to use dissolved organic phosphorus (DOP) as sole P source, in comparison to phosphate, over sustained multiple generations growth. The PO_4_ cultures represent a reference of growth under optimal conditions. We observe whether cultures provided with DOP instead of phosphate perform equally well in terms of growth rate and whether the cellular stoichiometry is altered. We closely monitored cell abundances, which we used to identify when, in the exponential phases, samples had to be taken. The medium used in these experiments initially contained 50 μmol P L^−1^, whether provided as an inorganic or organic form. Quantification of dissolved phosphorus in the cultures shows that, at the moment of sampling, there were from 25 to 30 μmol P L^−1^ left in the medium, which is a reasonable indication that cells had not consumed most of the phosphorus and so were not being limited by the availability of this element, ruling out the risk for some of the results to be biased by quantitative P limitation. Also, occurrence of any limitation would have shown as a rupture in the slope of the cell abundance vs. time. Figure 1 shows that we did sample before the growth curves taper off, indicating that all nutrients (micronutrients and vitamins, for instance) were still abundant; cultures were therefore unlimited by any nutrient at all times of sampling.

### Overall Growth Efficiency as a Function of the P Substrate for Growth

*Crocosphaera* grows on AMP and DL-α-glycerophosphate as also reported by Dyhrman and Haley (2006) but is unable to sustain the same growth rate on phytic acid. This latter result is in contradiction with the observations of Dyhrman and Haley (2006). All our cultures transferred in a medium with phytic acid as sole source of P just survive, indicating that cells are barely able to assimilate this compound and so may use it for growth over the long term, but in a very inefficient way. Our findings indicate that *Crocosphaera* cannot use all phosphomonoesters with the same efficiency, but it can grow without phosphate, provided that usable DOP is available. We obtain similar growth rates on phosphate or on dissolved organic phosphorus in the form of AMP or DL-α-glycerophosphate. All three showed a division time of about 3 days so each 30 day monitoring indicates that growth was sustained for a minimum of 10 generations. Figures 2, 3 in Dyhrman and Haley (2006) show that it takes at least 7 days of growth for a difference in population dynamics to be observed between cultures of *Crocosphaera* grown with or without phosphate. This suggests that cells grown without P may have kept growing for about a week using their internal P reserves. In their experiment, Pereira et al. (2019) followed cells for 144 h, which is 6 days. They observed induction of genes encoding high affinity transporters but did not observe the induction of genes that would allow for the synthesis of enzymes required for DOP use. Not knowing the actual magnitude of internal P reserves, we are thus unsure their experiment was carried out over a period long enough for cells to start sensing a severe limitation in their internal stock of Pi and to induce the gene regulation that would allow for DOP scavenging. So we do not rule out that cells grew on their internal reserves in their experiment.

The irradiance level applied here is saturating for this strain (Rabouille et al., 2017). When provided with sufficient light energy and non-limiting nutrients, *Crocosphaera* can thus grow equally well on some organic phosphorus as compared to phosphate. Still, we found a small, yet statistically significant difference in cell size between the treatments, with cells grown on DOP being larger than cells grown on phosphate (Table 1). Given that growth dynamics are similar in the three treatments, one could expect cells to divide at about the same rate, and so to have about the same size range. A bigger cell size may result from a slower growth rate. When cells are exposed to unfavorable conditions, their resource incorporation rate slows down and cells may not be ready at the time of division; they may thus miss the time window for division and, due to the tightly controlled cell cycle, only divide the following day (e.g., Dron et al., 2013). The time between two divisions increases, but then cells also experience a longer light exposure and a longer time to fix nitrogen, which results in a higher total C and N incorporation in between two divisions (e.g., Dron et al., 2013). Cells grown on organic P, because they are slightly bigger, must have somehow incorporated more material between two division phases, but did nevertheless divide at the same pace as the reference cultures grown on phosphate. The small difference observed between our treatments is not unusual as all of them remain within the range reported by Webb et al. (2009) in cultured *C. watsonii* WH8501 (2–4 μm) and also within that reported by Wilson et al. (2017) in natural *Crocosphaera* populations (2.1 ± 0.5 and 5.0 ± 0.8 μm for the small and large populations).

### How DOP Use Affects the Cellular Phosphorus Contents

Cultures grown on DOP do not present a systematic difference, in terms of C, N, and P contents, compared to the reference treatment grown on phosphate. Instead, the phosphate-grown cultures show a cell composition that is intermediate between the culture grown on AMP and that grown on DL-α-glycerophosphate. Cellular P contents are highest in the AMP culture, which is in agreement with the fact that AMP cells were slightly bigger. But the second highest P content is found in the reference culture grown on phosphate, and not in the second, largest-sized cells, meaning that phosphorus content is not the element making the difference in volume between the reference and the Alpha culture. This result also further supports the idea that the different phosphorus-containing molecules are not cleaved and uptaken with the same efficiency.

In all cultures, the phosphorus content, when expressed per cell, shows higher values in the early light phase and lower values in the late light phase (Tables 4 and 5). The similar trends in all treatments, with an increase during the dark phase and a decrease in the light suggest that the temporal acquisition dynamics remains unaffected whatever the phosphorus source. The occurrence of cell division in the mid light phase in this strain (Dron et al., 2012) creates a dilution effect as, upon mitosis, the content of a mother cell redistributes into the two daughter cells. We therefore estimated the P increase per cell in the dark, when cell size should not change as much. Not only does the phosphorus composition of cells vary between treatments, but so does the daily amplitude of the incorporation per cell (in the dark): +14% in AMP vs + 33% in the Alpha culture (Table 4). The response in the PO_4_ cultures falls in between the former two (+29%; Table 4), suggesting that inorganic phosphate does not necessarily yield the highest biomass when cultures are provided with sufficient energy (i.e., favorable irradiance conditions). Because cell size in both the AMP and Alpha culture is larger than in the reference, PO_4_ culture, the difference in P content could be due to a difference in cell size between experiments; we therefore expressed the phosphorus content per volume unit (Table 5). When normalized per cubic micrometer of cell, values become independent of a possible fluctuation in cell size with time, and so this avoids, in particular, the bias due to cell mitosis. This reveals an accentuated trend observed in the POP content compared to the per cell contents: The highest P content is observed in the AMP culture, with the lowest night increase (15%), and the lowest P content is observed in the Alpha culture, with the highest night increase (39%). The reference culture still shows intermediate P content and increase (29%).

Assuming the evolution of P content is monotonous during each light or dark phase, P appears to be rather incorporated at night and this daily dynamics is not modified whether P is inorganic or organic. If P is incorporated in the light, the per cell contents indicate that this incorporation is concealed behind the dilution effect due to cell division. Observation of the contents expressed per cell volume further shows that in the light, more phosphorus is lost than gained.

### Does the Alkaline Phosphatase Activity Reflect the DOP Use Efficiency?

Alkaline phosphatase activity was concomitantly recorded in all cultures. Activity in the reference PO_4_ culture remains very low or undetectable, only a few percent of what is observed in the Alpha culture, which shows the highest rates. The AMP culture shows a high activity although the normalized, recorded rates are two to six times lower than in the Alpha cultures. Alpha is also the culture with the lowest P content in cells. This could suggest that the activity of the alkaline phosphatase is proportional to the internal depletion in P. Yet, despite this enhanced activity, cells do not incorporate more P. Instead, it takes twice as high an alkaline phosphatase activity on DL-α-glycerophosphate for cells to incorporate equivalent amounts of P as compared to cells grown on AMP. The most plausible hypothesis is that the cleavage efficiency is not the same on all components, being lower on DL-α-glycerophosphate compared to AMP, which points to different efficiencies in scavenging compounds within the class of phosphomonoesters. This hypothesis is also supported by the measured, three-fold lower standing stock of phosphate in the Alpha cultures, which again suggests that alkaline phosphatase is less efficient in cleaving DL-α-glycerophosphate, than it is with AMP.

If the cleavage rate in the Alpha cultures is too low to meet the growth requirements, the liberated phosphate may very likely be consumed as quickly as it is produced and its concentration should remain close to undetectable, or at least at extremely low levels. The fact that soluble, reactive phosphate is clearly detected in the medium proves that the cleavage rate in the Alpha cultures, although lower, is still sufficient to cover the growth requirements. This result also corroborates well with the observed equivalent net growth rates between all three treatments. Note that we did not have a control for abiotic hydrolysis in the Alpha treatment, which, if not null, would also contribute to some (small) P supply. Last, the higher alkaline phosphatase activity in the Alpha culture did not yield a higher growth rate either. This result shows that neither P incorporation nor the growth rate are proportional to alkaline phosphatase activity. The latter is therefore likely a proxy for internal inorganic P depletion in line with PhoB regulation (Pereira et al., 2016). Alkaline phosphatase activity is thus not a suitable proxy to estimate DOP-based growth efficiencies of organisms, whether in culture experiments or in the natural environment.

### Impacts of DOP Use on the C and N Metabolism

In addition to testing the ability of *Crocosphaera* to grow on organic phosphorus only, we also aimed to tackle the question of the metabolic costs associated with the use of DOP, which may alter the cell stoichiometry. Ensuring a low contamination level in the cultures is thus crucial in many aspects. Part of the bacterial population would appear in the DOP pool and their growth would thus conceal the decrease in DOP concentration related to cyanobacterial uptake. Bacteria could also potentially consume the DOP and other nutrients, artificially enhancing the DOP decrease. The soundness of our results is further confirmed by the good correspondence between the POC and cell number increases that match the DOP decrease.

Cells perceive phosphate depletion in the medium as a stress that induces a suite of genetic regulations to first produce more of the high affinity phosphate transporters (Pereira et al., 2016) and then induce the production of the alkaline phosphatase to scavenge available DOP. We hypothesized that this chain of genetic and metabolic reactions would represent an energy cost for the overall metabolism, which may draw on the carbon reserves. That is, if there is a higher carbon cost due to DOP use, then the PO_4_ culture should show the lowest expenses and so possibly the highest POC content. But this is not what we observe here. In the present experiments, in which cultures are not limited by light, no significant difference in the C:N ratios appears between the phosphate-grown or DOP-grown cultures, meaning that using DOP did not result in imbalance on the carbon and nitrogen metabolisms. We also observe no significant difference in the absolute C or N contents in cells. These results indicate that if there indeed is a carbon cost to the use of DOP, the gross carbon acquisition efficiency was able to cover for it without visible impact on the net carbon incorporation in cells. Also, if DOP use imposes a carbon cost to cells, there could be competing carbon requirements in cells between the processes of N_2_ fixation and DOP use, which could induce a lower N_2_ fixation efficiency due to the redirection of carbon towards DOP scavenging. But the same conclusion applies to nitrogen: the genetic and metabolic processes required for the use of DOP were performed here without noticeable impact on the nitrogenase activity and on the net N acquisition in cells. So the overall gross carbon incorporation in all treatments was sufficient to support the carbon requirements of both processes of N_2_ fixation and DOP use. Also, contrary to what has been reported in *Trichodesmium* (Orchard et al., 2009), the equivalent nitrogenase dynamics and overall growth rates observed here suggest that nitrogenase synthesis is either not down-regulated when inorganic phosphate is not available, or the influx of phosphate derived from DOP scavenging prevented this downregulation to occur. Note that although they maintain about the same growth rate as the other treatments, the Alpha cultures tend to have a lower carbon content per cell. So their net carbon incorporation in between two divisions may have been slightly lower. This could be a hint for a higher carbon expenditure, if DL-α-glycerophosphate is not as easy to scavenge as AMP or phosphate.

Overall, the present results lead to the conclusion that *Crocosphaera* can sustainably grow on selected organic phosphorus as a sole phosphorus source at the same rates as on inorganic phosphate.

## Data Availability Statement

The original contributions presented in the study are included in the article/Supplementary Material, and further inquiries can be directed to the corresponding author.

## Author Contributions

SR, SD, PC, AL, and AO designed the study. LT conducted the experiments with contributions of AT, SR, and OC. SR and LT analyzed the data. SR drafted the manuscript and all authors provided input during writing of the manuscript. All authors contributed to the article and approved the submitted version.

## Funding

This work was supported by the French National program LEFE (Les Enveloppes Fluides et l’Environnement). SD was funded by NSF grants OCE 1434916 and 1737083.

## Conflict of Interest

The authors declare that the research was conducted in the absence of any commercial or financial relationships that could be construed as a potential conflict of interest.

## Publisher’s Note

All claims expressed in this article are solely those of the authors and do not necessarily represent those of their affiliated organizations, or those of the publisher, the editors and the reviewers. Any product that may be evaluated in this article, or claim that may be made by its manufacturer, is not guaranteed or endorsed by the publisher.
