# Supplementary material for: Organic Phosphorus Scavenging Supports Efficient Growth of Diazotrophic Cyanobacteria Under Phosphate Depletion
Source: Front Microbiol. 2022 Mar 25;13:848647. doi: 10.3389/fmicb.2022.848647 (PMC8990761; doi:10.3389/fmicb.2022.848647)
Supplement: Supplementary file 1 [file Data_Sheet_1.docx]

Supplementary Material


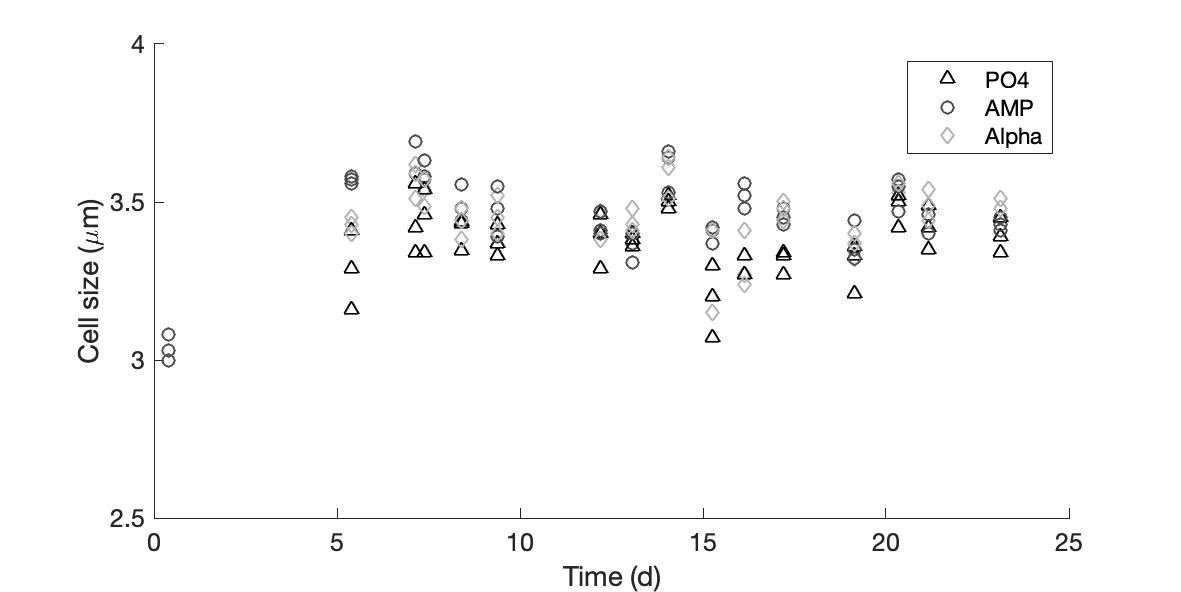


**Supplementary Figure 1.** Cell size (μm) measured in cultures provided with either an inorganic (PO4; triangles ) or organic (mono phosphate adenosin (AMP; circles) or DL-α-glycerophosphate (Alpha; diamonds)) source of phosphorus for growth. The period displayed here corresponds to the monitoring of cell abundances shown on Figure 1.


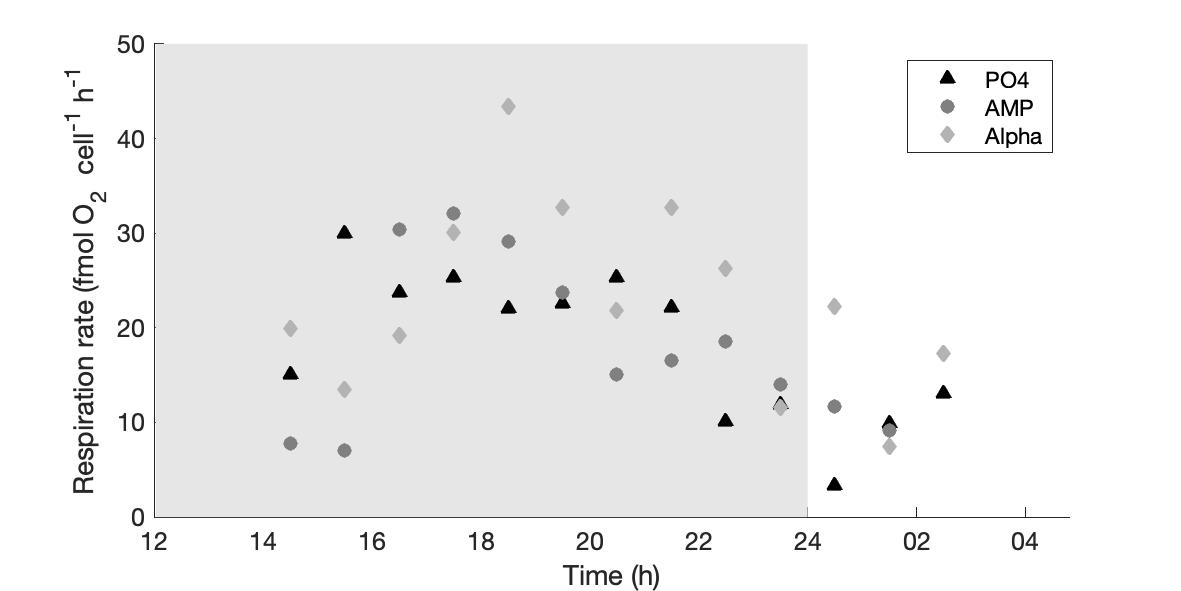


**Supplementary Figure 2.** Respiration rates (fmol O_2_ cell^-1^ h^-1^) measured over the dark phase in cultures provided with either an inorganic (PO4; triangles ) or organic (mono phosphate adenosin (AMP; circles) or DL-α-glycerophosphate (Alpha; diamonds)) source of phosphorus for growth. The grey, shaded area represents the dark phase.
